# Understanding barriers for refugees and migrants when accessing abortion care in Europe: a scoping review

**DOI:** 10.1186/s12889-026-27031-x

**Published:** 2026-03-21

**Authors:** Caroline Elfe, Talia Meer, Claudia Hövener, Stefanie Theuring

**Affiliations:** 1 Institute of International Health, Center for Global Health, Charité – Universitätsmedizin Berlin, corporate member of Freie Universität and Humboldt-Universität zu Berlin, Berlin, Germany; 2 Department of History, Humboldt Universität zu Berlin, Berlin, Germany; 3 Gender Health & Justice Research Unit, University of Cape Town, Cape Town, South Africa; 4 Alice Salomon University of Applied Sciences, Berlin, Germany

**Keywords:** Abortion care, Migrants, Refugees, Reproductive health, Barriers, Access, Patient-centred care

## Abstract

**Background:**

Access to safe abortion care has been the subject of international policy deliberation for several decades and was included in the World Health Organization’s essential healthcare services in 2020. It differs across Europe, with legal barriers like gestational age limits and the conscientious objection persisting in most European countries. Refugees and migrants can encounter additional barriers when accessing healthcare services for various reasons, such as health system exclusion and language barriers. The aim of this study was to map existing evidence on refugees’ and migrants’ access to abortion care in Europe.

**Methods:**

Adopting a scoping review methodology, we conducted a systematic search in PubMed, complemented by web-based hand searches and citation tracking to identify the relevant literature. We included qualitative and quantitative studies as well as grey literature published in English between 2014 and 2025, addressing how refugees and migrants accessed abortion services across Europe. Data were charted according to Levesque et al.’s dimensions of healthcare access framework. Results were reported using a narrative synthesis approach.

**Results:**

We identified 19 studies in eleven different European countries and one EU-wide report (*n* = 20), including twelve qualitative, five mixed, and three quantitative designs. While some studies focused specifically on migrant women seeking reproductive healthcare, others focused on abortion seekers in general while highlighting barriers unique to migrant populations. Prominent barriers were lack of information and insufficient availability of translation services. Availability of services was often restricted by regional disparities and restrictive laws. Affordability issues were amplified by variations in cost entitlements and the precarious status of undocumented migrants. Quality of care was compromised by discriminatory attitudes held by providers.

**Conclusions:**

Refugees and migrants in Europe face barriers in access to abortion tied to their migration context. Gaps in coverage for refugees and migrants constitute a violation of universal human rights. Ensuring access requires improved outreach efforts, free availability of translation services, decriminalization, and integration of abortion care into a Universal Health Coverage framework, while combating racism in health care and amplifying the voices of marginalized groups.

**Supplementary Information:**

The online version contains supplementary material available at 10.1186/s12889-026-27031-x.

## Introduction

Access to safe abortion care has been the subject of international policy deliberation since the 1970s. The 1994 Programme of Action of the International Conference on Population and Development in Cairo framed unsafe abortion primarily as a public health issue and urged states to ensure safe services where abortion is legal [1]. In 2020, the World Health Organization included comprehensive abortion care in its guidance on essential health services, further consolidating its status within global health policy. Globally, 61% of all unintended pregnancies end in induced abortion. Thus, abortion is a common procedure, which is low risk when performed safely [2]. When compared to the general population in European countries, rates of unintended pregnancies tend to be higher among refugees, recent and undocumented migrants, who have an increased risk of being exposed to sexualized violence and more often have limited access to contraceptives [3–6]. According to the UN Refugee Agency (UNHCR), Europe is expected to host over 13 million refugees in 2025, making it one of the regions with the highest number of displaced people globally [7]. In 2024, 4.3 million people migrated to the European Union (EU) from non-European countries, which is a decline in 18% compared to the year before [8]. Data on the exact number of undocumented migrants in Europe is not available as many cases remain unreported. Recent estimates count between 2.6 and 3.2 million undocumented migrants in twelve European countries over the period 2016 and 2023 [9].

In response to the overturning of *Roe v. Wade* by the US Supreme Court in 2023, the European Parliament (EP) has repeatedly urged member states to decriminalize abortion and include the right to abortion in the EU Charter of Fundamental Rights [10, 11]. However, since the issue of abortion falls under the principle of subsidiarity, member states regulate it independently through national legislations [10, 11]. As shown in the European Policies Abortion Atlas published by the European Parliamentary Forum for Sexual and Reproductive Rights (EPF), which scored European countries and territories on legal frameworks for safe abortion care, these differ greatly across Europe [12]. National laws and health systems influence both access and the methods available for pregnancy termination [13]. Most European countries allow abortion on request or on broad grounds, but few grant unrestricted access to abortion care [14]. Even in countries where abortion is available on request, regulations such as gestational age (GA) limits, obligatory counselling, or the option for physicians to conscientiously refuse care limit and complicate access to care for abortion-seekers [14–16]. As Fiala et al. reported, this heterogeneity indicates that abortion legislation is neither based on scientific evidence nor on the needs of abortion-seekers [14]. Depending on national policies, European citizens face significant legal and regulatory barriers when accessing safe abortion care. For refugees as well as recent and undocumented migrants, access to safe abortion services can be even more complicated due to their limited access to the national health systems.

Healthcare services in European countries are often hard to access for refugees and migrants for administrative, legal, cultural, linguistic, financial and many other reasons [17–19]. Multiple studies have highlighted the need for developing structures at the EU level which offer long term solutions for providing inclusive healthcare at all stages of the migration trajectory to refugees and migrants instead of chaotic emergency interventions [17, 20]. Previous studies have shown that refugees and migrants face additional hurdles when accessing sexual and reproductive health (SRH) services and have significantly worse SRH outcomes compared to the general population in high income countries, which is sometimes linked to their underutilization of SRH services [3, 4, 21–23]. A 2024 systematic review on undocumented migrants’ access to SRH care found that existing barriers include refusal of care, lack of knowledge about national healthcare schemes, fear of deportation, bureaucratic hurdles, and affordability issues [19]. Another 2024 systematic review which reported on abortion experiences and perspectives amongst migrants and refugees, concluded that abortion care must account for patients’ cultural and social understandings of reproduction and pregnancy to ensure equitable access [24]. However, as previous reviews indicated, most studies on SRH of migrants and refugees focus mainly on prenatal and perinatal care while research on access to safe abortion services remains scarce [19, 25]. We chose to focus on the whole of Europe including EU member states, European Free Trade Association (EFTA) member states, and the United Kingdom (UK), since ecologies of exclusion are often similar within different legislations and national contexts. Central research questions that are not answered yet include: What are the key barriers faced by refugees and migrants when accessing safe abortion care in Europe? Which informational, linguistic, financial, logistic, and administrative hurdles contribute to disparities in abortion care access? How is the accessibility of abortion services influenced by the intersection of healthcare systems, migration status, and legal restrictions? The aim of this study was to contribute to answering those questions by mapping existing evidence on refugees’ and migrants’ access to abortion care in Europe.

## Methods

This study draws on the scoping review methodology developed by Arksey and O’Malley [26] which was further refined by Levac et al. [27] and provides recommendations for each subsequent stage of the research process: identifying the research question, searching for relevant studies, selecting studies, charting the data, collating, summarizing, and reporting the results. While this methodology provided the cornerstone for our research, we sometimes deviated from the recommended order of steps due to practical constraints. The study design was chosen to accommodate the broad scope of our research objective, enabling the inclusion of studies from diverse European contexts, employing different methods and drawing on various types of data sources [28]. For reporting, we used PRISMA guidance [29]. We did not publish or register our study protocol.

### Definitions & terminology

Drawing on the definition by the International Organization for Migration (IOM), migrants are defined as people who “move away from [their] place of usual residence, whether within a country or across an international border, temporarily or permanently, and for a variety of reasons.” [30]. Refugees are defined in accordance with the UNHCR as people who are “forced to flee their own country and seek safety in another country” [7]. Respective definitions of migrants and refugees adopted in the included studies may differ from our definitions. It is important to note here that migrants are a highly diverse group, and that our findings may be more applicable to recent and undocumented migrants than other migrant groups. Furthermore, we recognize that not all individuals seeking abortion care identify as women. However, in most studies that we examined for this review, refugees and migrants who were seeking abortion care were described as women. While trying to refrain from gendered language as much as possible, we will sometimes refer to participants in these studies as women.

### Search strategy and selection criteria

**Fig. 1:**
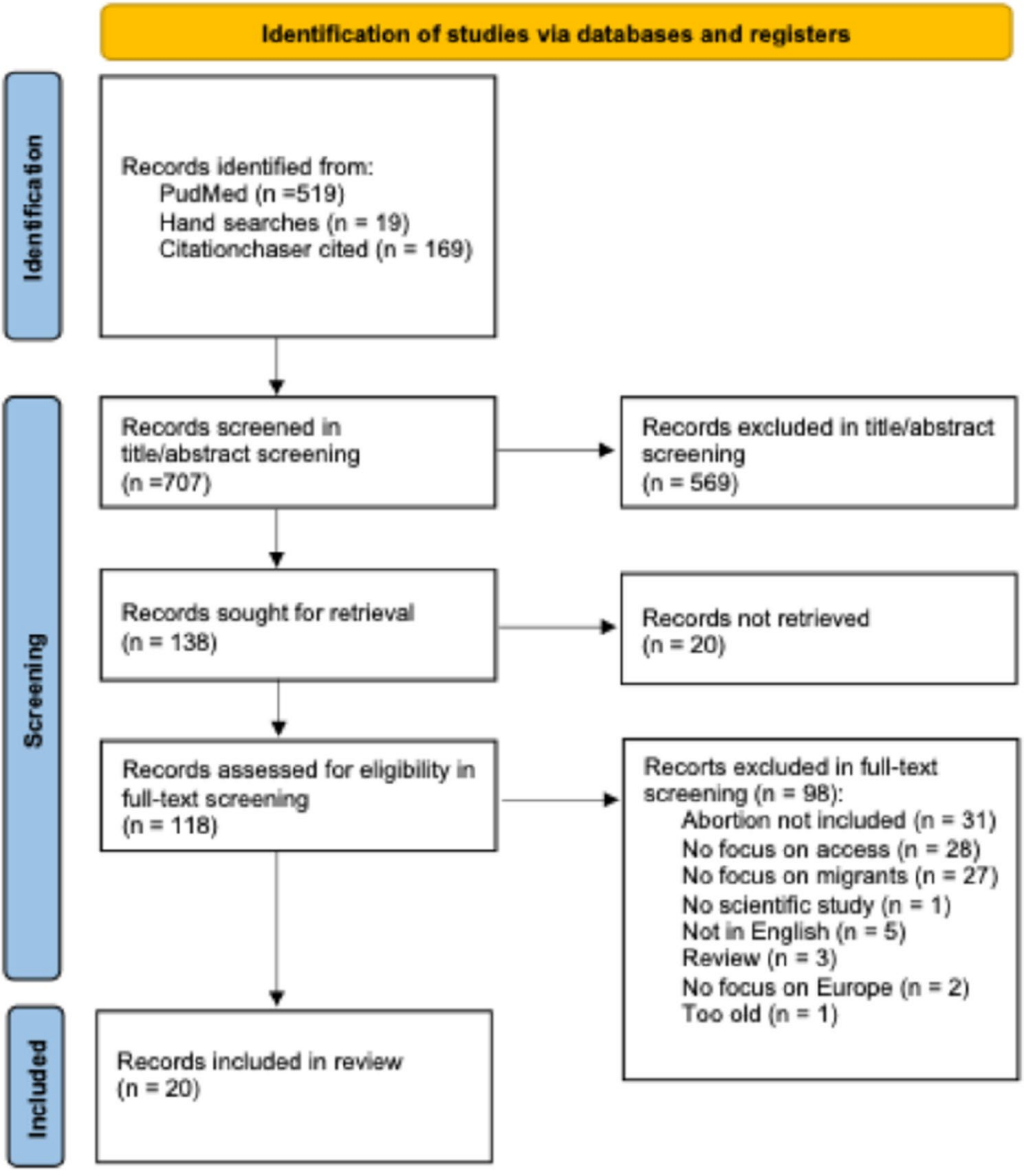
PRISMA flowchart

The researchers defined the primary research objectives, specified search terms, selected databases for the literature search, and established eligibility criteria according to the Population Concept Context (PCC) framework [28]. Studies were selected if they included refugees or migrants in the study *population* and/or study focus group, addressed access to abortion as a *concept*, were conducted within the *context* of Europe, i.e. EU member states, EFTA member states, and the UK, were published in English, and were published from 2014 onwards. We included non-EU countries (i.e., EFTA members and the UK) because the social and institutional dynamics relevant to our research often transcend EU-specific legislation. A comprehensive literature search was conducted in PubMed. The database search was complemented by web-based hand searches were as well as a combination of direct backward and forward citation tracking [31]. Search terms were selected to encompass a range of relevant elements, and included (“abortion” OR “reproductive health” OR “SRH”) AND (“refugee*” OR “asylum seeker*” OR “migrant*”) AND (“Europe” OR “EU”) (see Appendix A for the complete search string). Relevant evidence was identified using a four-step process which is illustrated in Fig. 1. The screening was conducted by a single researcher. We limited our search to peer-reviewed articles and grey literature published in English between 2014 and 2025 in order to keep results topical, given changes in policies and legislations. The latest search was carried out in April 2025.

### Data charting and synthesis of results

The selection process (illustrated in Fig. 1) resulted in a total of 20 studies for the final review. Study focuses differed with some looking at migrant women seeking SRH care, while others were targeting abortion-seekers more broadly, but addressed specific hurdles faced by migrant groups; we emphasized themes specifically pertaining to migrant/refugee-specific barriers. We charted data from the included studies into a self-developed extraction tool, focusing on characteristics such as the authors, publication year, setting, methodology, participants and study objective. In the next step, the new categories were added to the charting sheet based on Levesque et al.’s framework of patient-centered healthcare access, looking at access to abortion care through the dimensions of approachability, acceptability, availability, affordability and appropriateness [32]. Drawing on this framework, we defined approachability in terms of individuals’ ability to recognize the existence and relevance of health services, which is influenced by factors such as transparency, outreach activities, and health literacy [32]. Acceptability was understood as the cultural and social alignment of services with patients’ beliefs and values which affects their willingness to seek care [32]. Availability was defined as the physical presence and timely provision of health services which depends on factors such as infrastructures, service distribution, and mobility [32]. Affordability was defined as individuals’ economic capacity to pay for healthcare, including direct costs, opportunity costs, and the ability to mobilize resources without sacrificing basic needs [32]. Finally, appropriateness was understood as the alignment of healthcare services with individuals’ needs, encompassing the quality of care as well as the potential for patients’ engagement in the care process [32]. We chose this framework for its comprehensive and multilevel conceptualization of access along different stages in the process of seeking and receiving care. We conducted a content analysis of the 20 selected publications, i.e. key data in the publications were coded according to the charting categories and extracted into the tool. A narrative synthesis approach was used to present the findings. The results section is structured to follow Levesque et al.’s framework [32].

## Results

### Search results

This review identified 20 studies which addressed abortion care access for migrants and refugees in Belgium [33], the EU [34], Denmark [35], Germany [36, 37], Greece [38], Ireland [39–41], Italy [38], the Netherlands [42], Spain [38, 43–45], Sweden [46–49], Switzerland [50, 51], and the UK [52]. Methodologies varied across studies and included twelve qualitative [33, 35, 38–40, 42–46, 48, 51], five mixed [34, 36, 37, 41, 50] and three quantitative designs [47, 49, 52]. The studies were published between 2016 and 2025. An overview of the included studies is shown in Table 1.

**Table 1:** Included studies

Author(s), Year	Setting	Information source	Study focus group	Methods	Study purpose	Approachability	Acceptability	Availability	Affordability	Appropriateness
Åkerman et al. 2016 [49]	Sweden	Thai women who immigrated to Sweden (*n* = 804)	Thai women who immigrated to Sweden	Survey	Investigate knowledge and utilization of SRH services among Thai immigrant women	X				
Amroussia 2022 [46]	Sweden	Providers working in youth clinics & women healthcare clinics (*n* = 31)	Migrants	Semi-structured interviews	Explore providers’ challenges & navigation strategies when providing SRH to migrants	X	X	X	X	X
Chakravarty et al. 2023 [39]	Ireland	Service users who sought abortion care (*n* = 30)	Service users of abortion care	Qualitative in-depth interviews	Provide a comprehensive understanding of service user experiences with abortion care	X	X	X	X	X
Cignacco et al. 2018 [50]	Switzerland	Health & social care Professionals (*n* = 9)	Women asylum-seekers	Semi-structured interviews & survey	Assess healthcare provision & to what extent it addresses SRH needs	X	X	X	X	X
De Kort et al. 2021 [33]	Belgium	Staff at abortion centre (*n* = 11)	Abortion-seekers	Semi-structured in-depth interviews	Describe how Covid-19 measures affected quality of care		X			
Duffy et al. 2022 [41]	Ireland	Quantitative data, web-based information, & qualitative data from interviews with providers (*n* = 51), key informants (*n* = 27) and service users (*n* = 30)	Abortion-seekers	Patient Journey Analysis	Interrogate how information flow stratifies access to abortion care	X		X		X
EIGE 2024 [34]	EU	Questionnaire: Experts (*n* = 26), Interviews: Representatives from relevant organisations (*n* = 12)	Women & girls fleeing the war under Directive 2001/55/EC	Questionnaire & qualitative interviews	Assess access to SRH services essential in clinical management of sexual violence	X	X	X		X
Grotti et al. 2018 [38]	EU borderlands (Greece, Spain, Italy)	Migrant women & healthcare professionals	Pregnant migrants entering the EU via Mediterranean borders	Ethnographic research: participant observation & interviews	Examine pregnant migrants’ experiences of reproductive care in EU borderlands		X	X		X
Fern 2025[44]	Spain	Immigrant women who sought SRH care (*n* = 69)	Immigrant women seeking SRH care	Semi-structured interviews	Unpack how immigrant women experience harm in SRH care					X
Holten et al. 2021 [42]	Netherlands	Women who had an abortion (*n* = 20), providers (*n* = 14) and women seeking abortion through WoW (*n* = 200)	Abortion-seekers	Semi-structured Interviews & qualitative analysis	Identify key barriers encountered by abortion-seekers	X	X	X	X	X
Jones et al. 2021 [52]	UK	Clinicians (*n* = 343)	Migrants	Survey	Explore clinicians’ knowledge of healthcare charging regulations and terminology	X			X	
Killinger et al. 2022 [36]	Germany	Online consultations with WoW (*n* = 1048) & emails (*n* = 108)	Women consulting WoW	Cross-sectional analysis & content analysis	Identify key barriers encountered by abortion-seekers	X	X	X	X	X
Larsson et al. 2016 [48]	Sweden	Midwives (*n* = 10) & doctors (*n* = 3)	Immigrant women	Qualitative interviews	Explore providers’ experiences with providing abortion care to immigrant women		X			X
Marti Castaner et al. 2021 [35]	Denmark	SRH care providers outside formal healthcare sector (*n* = 6)	Undocumented migrants	Semi-structured interviews	Explore the tactics adopted by healthcare providers in the humanitarian aid sector to meet SRH needs of undocumented immigrant women	X	X	X		X
Martín 2016 [45]	Madrid, Spain	Abortion-seekers and providers in Madrid	Migrant women	Ethnographic fieldwork drawn from own experience as GP in program for undocumented migrants	Explore how abortion legislation & policies are implemented in public healthcare			X	X	X
Mishtal et al. 2022 [40]	Ireland	Providers (*n* = 22), service users (*n* = 30), key informants (*n* = 27)	Service users and providers	Qualitative in-depth interviews	Examine the barriers and facilitators of the Irish abortion policy implementation		X	X	X	
Ostrach 2020 [43]	Catalunya, Spain	Women seeking abortions at fieldwork clinic (*n* = 28)	Migrant & low-income women seeking abortions	Participant observation & informal interviews	Assess continuity and changes of publicly funded abortion care			X		
Rød et al. 2023 [37]	Germany	Online consultation form (*n* = 2057), Health practitioners (*n* = 8)	Women consulting WoW	Survey & Qualitative interviews	Explore abortion access during the Covid-19 pandemic		X	X	X	
Schmidt et al. 2018 [51]	Geneva, Switzerland	Migrant women (*n* = 78)	Migrant women	Focus group interviews (*n* = 13)	Explore barriers to reproductive health services faced by migrant women	X	X		X	X
Tirado et al. 2023 [47]	Sweden	Recently arrived migrants attending high schools or Swedish language schools (*n* = 6263)	Recently arrived migrants	Survey	Investigate migrants’ knowledge on the right to safe and legal abortion and other associated factors	X				

### Approachability

Hurdles relating to approachability were reported in twelve studies [34–36, 39, 41, 42, 46, 47, 49–52]. Studies from the EU, Ireland, Netherlands, Sweden and Switzerland identified informational barriers at point of entry as an access barrier for some migrant groups who had limited knowledge of their options, had difficulties navigating the healthcare system and were more vulnerable to misinformation [34, 41, 42, 46, 49, 51]. Abortion-seekers across the EU reported challenges in finding neutral scientific information online and identifying suitable providers [34, 42]. Studies from Switzerland and the UK suggested that healthcare workers themselves lacked knowledge on the health needs and entitlements of asylum-seekers and migrants [50, 52]. Fear of authorities was reported as an access barrier in studies from Belgium, Denmark, Germany, Netherlands and Switzerland [35, 36, 42, 50, 51], especially among undocumented migrants who were reluctant to seek care due to fear of deportation [35, 36, 42]. One study observed that asylum-seekers who were in transit from Italy to Switzerland were discarding their medical documents and medications to avoid being registered in Italy [50]. Furthermore, a study from Sweden found that nearly three-quarters (74%) of recently arrived migrants did not know that abortion is legal in Sweden, suggesting massive deficits in health literacy on the right to abortion care [47].

### Acceptability

Barriers relating to acceptability were identified in thirteen studies [33–40, 42, 46, 48, 50, 51]. Studies from Belgium, the EU, Sweden, and Switzerland identified a preference for a healthcare provider of the same gender among migrants seeking SRH care [34, 46, 50, 51]. However, as noted by a study on access of Ukrainian girls and women to SRH care after having been victims of sexualized violence, only seven out of 26 Member States made it obligatory to comply with a patient’s preference for a female professional in SRH services [34]. Another barrier relating to acceptability which was identified by studies from Germany, Netherlands, and Sweden was the need for secrecy [37, 42, 48]. A study from the Netherlands which examined motivations of abortion-seekers resorting to a provider outside the formal healthcare sector identified secrecy as imperative for migrants’ preference for self-managed abortion [42]. A study from Switzerland reported embarrassment and discomfort as personal barriers to pelvic and vaginal examinations, which were sometimes related to providers’ missing awareness for a patient’s cultural traditions [51]. Furthermore, some providers in Sweden perceived limited knowledge of anatomy, reproduction and contraception among some migrant women as a barrier during consultations [48].

Across most countries, another central hurdle relating to acceptability was dealing with language barriers [33–40, 42, 46, 48, 50, 51]. Interpreters were not always available in the language of the patient [34, 37, 40, 42, 46] and lacked knowledge on the necessary medical terminology and expertise in communicating sexuality-related issues in a sensitive way [40, 46]. In the absence of professional interpreters, providers in Ireland had to use Google Translate or had a relative, friend or colleague of the patient translate for them which is against clinical guidance [40]. In Switzerland, providers had to resort to creative methods like gesturing, acting or drawing in order to facilitate communication [50]. If available, interpreters were typically only present during planned consultations with physicians, but not for interactions with receptionists or in emergencies [42, 46]. A study conducted in the Netherlands found that although young migrants preferred medical abortion due to its alignment with their need for discretion, proficiency in Dutch or English was a prerequisite for access, as communication in the event of complications was only possible in those languages [42]. Furthermore, a study from Sweden noted that language difficulties were exacerbated by the time constraint of the encounter even with an interpreter present [46]. A study from Belgium found that Covid-19 regulations further complicated language barriers as wearing masks and online consultations made non-verbal communication and building trust more difficult [33].

### Availability

Thirteen studies reported on hurdles relating to availability [34–43, 45, 46, 50]. The literature highlighted regional disparities in both the availability of abortion providers and regulatory access for refugees and migrants, noting that central urban areas generally exhibit a higher concentration of providers, while rural and suburban areas – where asylum-seekers are commonly accommodated – remain comparatively underserved [37, 39, 45, 46, 50]. Moreover, a study from Ireland reported that accommodations for asylum-seekers were unsuitable for maintaining privacy during GP visits or for self-administering early medical abortions [39]. Studies from Germany and the Netherlands which looked at the reasons why abortion-seekers consulted with Women on Web (WoW), an international non-governmental organization (NGO) providing telehealth abortion outside the formal healthcare system, identified logistic barriers such as waiting times, transportation, bureaucracy, and obtaining the right form of documentation (such as proof of health insurance or residence permit) as central access barriers [36, 42]. A report on access barriers faced by Ukrainian refugees seeking abortion care revealed that only three out of 26 EU member states (Austria, Denmark and Sweden) made abortion care available without documentation [34]. Furthermore, a study on access to abortion care in Catalunya, Spain, found that migrant women had to make more visits and had longer waiting times compared to locally born women [43].

Restrictive legislation was identified as a barrier limiting availability in multiple countries [34, 38, 40, 41]. In most EU countries, abortion was granted only in exceptional cases or tied to specific conditions which are additional burdens for abortion-seekers who are unfamiliar with the national health system [34]. Conscientious refusal of care, obligatory consultations, mandatory waiting times, and GA limits were identified as legal barriers [34, 38, 40, 41]. The difficulties that can arise were illustrated by a study on pregnant migrants’ experiences of reproductive care in the EU borderlands: Italian law required abortion requests to be made to a gynecologist within the first three months of pregnancy, limiting access for migrants arriving when the pregnancy was more advanced, while access was further hindered in Sicily, where 87.6% of doctors refused to provide care based on the conscientious objection [38]. Some studies reported that the limited availability of abortion care within the public health system meant that NGOs had to step in to fill this gap [35, 36].

### Affordability

Ten studies highlighted hurdles relating to affordability [36, 37, 39, 40, 42, 45, 46, 50–52]. Cost entitlements for abortion care were found to differ depending on country, region, migration status and other criteria [36, 40, 46]. In studies from Ireland and Spain, cost coverage for abortion care was only granted to those with a social security number or health insurance card, posing unique challenges to migrants who had recently arrived or were undocumented [39, 40, 45]. Some studies identified an insufficient awareness of cost entitlements and support initiatives among migrants or refugees as well as among health professionals themselves as a barrier [34, 46, 52]. Gaps in coverage also represented a dilemma for health professionals who wanted to provide care for those in need [40, 46]. Undocumented migrants were identified as the subgroup most affected by financial barriers as they were more often not eligible for cost coverage and inhibited by the fear of having their migration status revealed [36, 42, 46]. A special risk for suffering financial hardships was found in undocumented adolescents [37]. In countries with poor online infrastructure, getting cost coverage for abortion became more difficult during the Covid-19 pandemic [37]. Furthermore, indirect costs, i.e. transport, organizing childcare, and taking a day off from work were identified as common barriers in the literature [36, 37, 39, 45, 50].

### Appropriateness

While we found no indication of low quality in the safety or effectiveness of methods used for pregnancy termination in the literature, twelve studies indicated quality deficits in the larger care delivery process [34–36, 38, 39, 41, 42, 44–46, 48, 50, 51]. A study from Switzerland reported that asylum-seekers had to undergo medical exams repeatedly because medical information from previous exams was missing [50]. Multiple studies indicated that health professionals lacked expertise and guidelines in providing gender and diversity-sensitive care to refugees and migrants [34, 35, 46, 50]. Two studies focusing on experiences of providers in Sweden pointed out that providers perceived migrant patients from different cultural backgrounds as passive and found it challenging to engage them during the healthcare encounter [46, 48]. The literature also indicated that quality of care suffered from racist attitudes held by health professionals resulting in impolite treatment and longer waiting times [42, 46, 51]. A study reporting on the experiences of pregnant migrants from Syria who received care in Athens argued that healthcare provision was shaped by cultural and gendered stereotypes [38]. A study from Spain on immigrant women’s experiences of harm in SRH care reported that health professionals expressed dismissive attitudes, inappropriate behavior and racist attitudes as well as failing to respect patient autonomy and mistreating patients [44].

Additionally, studies from Denmark and Spain indicated that migrants without health insurance were dependent on the knowledge and goodwill of individual healthcare professionals, leaving them in an especially precarious position [35, 45]. Further, abortion stigma was found to impede high quality care, manifesting in negative provider attitudes, judgmental comments from healthcare professionals, delays in care provision, lack of assistance, and harassment in front of clinics [34, 36, 39, 41, 42]. Abortion stigma was also associated with internal barriers, making it more difficult to talk about the wish for an abortion and seek help [34, 42].

## Discussion

This scoping review aimed to create an overview of barriers in access to abortion care in Europe faced by refugees and migrants, based on Levesque et al.’s framework of patient-centered access. The reviewed literature indicates that massive access barriers persist in all five access dimensions. Barriers were found in different regional and national settings across Europe, often specific to the refugee-/migrant-contexts. Migrants and refugees are highly heterogenous groups, and access varied according to personal resources, social identities, perceived identities within clinical encounters, and the broader structural and living conditions. Legal status emerged as key determinant among migrants/refugees as absence of residence permit was directly linked to the deprivation of care.

Central hurdles identified in this review include a lack of knowledge on entitlements among refugees and migrants as well as difficulties navigating the healthcare system and identifying suitable providers. These findings point to major deficits in transparency, outreach activities and available information on abortion care. While similar barriers also exist for other types of healthcare services and have previously been linked to migrants’ and refugees’ underutilization of primary healthcare services [53], our findings suggest that the stigmatized and legally restrictive context of abortion provision exacerbates difficulties in retrieving information on providers and entitlements.

Furthermore, our results demonstrate that undocumented migrants’ access is constrained by fears of being reported to immigration authorities by healthcare professionals, which reflects a broader distrust of public institutions. Previous instances of data sharing between healthcare providers and immigration authorities in the UK show that this distrust is not unfounded [54]. Taking up the issue of trust in their approach to migrant-sensitive healthcare, Savas et al. have warned that trust in this context must be “earned and maintained, and can easily be undermined and hampered” [17]. They consider participatory approaches and community engagement in the provision of healthcare to be the most effective ways of re-gaining the trust of undocumented migrants [17]. A study from 2019 further recommended the use of community health educators and language-appropriate written material to improve access to information [20].

Our findings indicate that insufficient availability of diversity-sensitive and linguistically appropriate care further constrains access. Care seekers’ preference for a female physician [34, 46, 50] and the need for secrecy [37, 42, 48] were identified by some studies as “cultural barriers”. However, as similar preferences have been observed in studies on the general population and these preferences are thus not exclusive to migrant populations, we refrain from discussing them as a migrant-specific finding [55]. The lack of skilled interpretors, on the other hand, which was reported as a hurdle by numerous studies, represent a hurdle disproportionately affecting migrants and refugees [34–37, 42, 46, 50]. This finding is consistent with existing evidence repeatedly stressing the importance of translation services for ensuring equitable access [56, 57]. A study on self-reported healthcare discrimination and the availability of translation services suggests that linguistically diverse healthcare services should be one of the main aims of relevant health policies and strategies at the European level in order to respond to the unmet needs of migrant populations [56]. The same study also found gender inequalities in access, indicating that female migrants are more likely than their male counterparts to have unmet healthcare needs [56]. A previous review which synthesized qualitative data on irregular migrant women’s experiences of SRH care similarly highlighted the need to reduce language barriers, while also arguing that the use of interpreters can result in a lack of confidentiality leading to migrant women feeling distrustful [57]. It is therefore pivotal to make sure that the use of translation services does not conflict with the need for secrecy.

Our results show that offering linguistically appropriate care requires organizational shifts which go beyond the presence of an interpreter during consultations. A study from Sweden argued that physicians need to schedule more time for consultations with patients who require translations and insisted on qualified interpreters to be present beyond the planned encounter with the physician (i.e., for scheduling appointments) [46]. Another solution for overcoming both cultural and linguistic barriers proposed by a study from Denmark was finding providers who share the linguistic and cultural background of the patients [35]. In contrast to that, a previous review on language and cultural barriers and facilitators of SRH care for migrant women argues that it is essential for providers to focus not on women’s “cultural backgrounds” but on applying an empathetic gaze as well as a willingness to listen to a woman’s SRH needs and preferences and to try to accommodate them [58].

Unsurprisingly, our study revealed that refugees and migrants were disproportionately affected by bureaucratic hurdles, logistical barriers and restrictive legislation on abortion [37, 46, 50]. While gaps in the availability of abortion care like regional differences in providers, complicated appointment mechanisms, obligatory counseling, conscientious refusal of care and GA limits pose challenges to abortion-seekers regardless of their citizenship, migrants and refugees can be affected by these hurdles more severely. As a study on reproductive care in the Meditarreanean border regions of the EU as well as studies from Ireland and Switzerland have shown, asylum-seekers living in reception centers are often cut-off from public transport and are limited in their mobility [38, 39, 50]. Organizing travel to the next abortion clinic can thus constitute a substantial barrier, especially if looked at in combination with previously discussed hurdles such as language barriers and lack of information. The example of GA limits illustrates how refugees and migrants are affected more severely by restrictive legislation than abortion-seekers who are European citizens. The migratory context may result in delays in care, making it more likely that GA limits are passed, especially for people coming to Europe by boat who often first arrive on islands where abortion care is not available [38]. Furthermore, once GA limits have passed, refugees and migrants whose freedom of movement is curtailed by travel restrictions may not have the option to travel abroad for a second trimester abortion. Therefore, as de Zordo et al. have argued previously, GA limits are not just a limitation of reproductive freedom but also a matter of reproductive justice [15]. Studies included in this review have indicated that the NGO-based distribution of medical abortion pills can be a solution to bypass restrictive legislation and logistic hurdles at least for abortions during the first trimester [36, 37, 42]. A recent review by Napier-Raman et al. has noted that there is little discussion of self-managed abortion outside unsafe abortion methods, leaving room for future research [24].

We found gaps in cost coverage to be significant access barriers, affecting undocumented migrants the most [42, 46]. On the one hand, affordability problems are linked to the criminalization of abortion in some European countries. Criminalization leads to regulation of abortion care separately from other types of healthcare services, and to out-of-pocket payments which can only be reimbursed retrospectively (if reimbursement schemes exist at all). On the other hand, gaps in coverage are also linked to migration status. Despite the EU’s commitment to the UN Sustainable Development Goals which demand the implementation of Universal Healthcare Coverage (UHC) by 2030 for everyone regardless of their migration status, most European countries have not extended coverage to undocumented migrants [54]. At the same time, the EU’s decision to create access to healthcare for Ukrainian citizens under the Temporary Protection Directive 2001/55/EC has shown that extending coverage to migrants is possible where political willingness exists [54]. While in practice, access to SRH care for Ukrainian citizens has not been fully realized due to the persistence of major hurdles such as language barriers, this decision was a major step [34]. However, as previous research has noted, the differential treatment of Ukrainian citizens compared to Ukrainian war refugees with other nationalities or compared to refugees from other conflict zones like Syria and Afghanistan stands in stark opposition to principles of UHC and universal human rights [17].

Another notable finding of this review is that quality deficits in care delivery often resulted from racist attitudes and gendered stereotypes held by healthcare professionals [38, 42, 46, 48]. This is consistent with previous research which found that racialized minority healthcare users experience overt and covert forms of racism from healthcare providers, i.e. being dismissed, devalued or left out from decision-making processes [58, 59]. As described in the study by Grotti et al., the gendered racism experienced by refugee women in the context of reproductive care in Greek hospitals can manifest in perceptions of them as “extremely vulnerable”, further exacerbated by language barriers [38]. African women were typically seen as victims of sexual exploitation and Syrian women as deprived of reproductive agency and lacking knowledge of their own bodies [38]. While emphasizing the real vulnerability of refugee women due to the circumstances of their journey, Grotti et al. criticize the concept of vulnerability for its paternalistic undertone, which can deprive women of agency in care encounters [38]. The concept has also been criticized elsewhere for focusing on the individual and shifting the attention away from structural causes like migration processes and legislation [60]. An intersectional approach can help understanding this type of gendered racism which frames racialized women as passive and oppressed [61]. Conceptualizing different forms of oppression, i.e. racism and sexism, not as additive but as interlinked systems where “lived identities, structural systems, sites of marginalization, forms of power, and modes of resistance ‘intersect’ in dynamic ways”, as described by Vivian M. May, intersectionality is useful for understanding the realities of people who are marginalized at multiple levels [62]. Improving access will thus require an integrative approach which addresses the interplay of multiple barriers rather than looking at each barrier separately.

Overall, the access barriers identified in this review illustrate that refugees and migrants were affected by two interlinked forms of criminalization: the criminalization of abortion which limited access to abortion care through restrictive laws, and the criminalization of migration which excluded certain migrant groups from coverage. Our findings suggest that abortion care in European countries does not sufficiently address the needs of migrants and refugees. As a result, some pregnant individuals may attempt unsafe methods for self-induced abortion or be forced to continue an unwanted pregnancy, which can have detrimental effects on their physical and mental health [63].

### Limitations

This review has several limitations. Since there was very little evidence on the topic, we used broad inclusion criteria resulting in significant variability across the included studies, making direct comparisons between individual studies difficult. The findings are not representative of the diverse policy landscape in Europe since Northern European countries like Sweden and Ireland were overrepresented while Eastern European countries were only represented in the one EU-wide study. Furthermore, refugees and migrants are broad categories including a wide range of persons whose experiences differ enormously, depending on their respective social, economic and personal realities. A restriction to English-language publications may have led to the exclusion of important studies published in other languages. Also, the perspectives of refugees and migrants were not central to most of the reviewed literature, as many studies focused on experiences of providers and their perceptions of patients who were either refugees or migrants. Whether their assessment overlaps with the first-hand experiences of refugees and migrants and to what extent providers’ perceptions were shaped by cultural and gendered stereotypes cannot be ascertained. Our results should thus be interpreted with caution.

The fact that only 20 studies were identified since 2014 in all of Europe speaks to a significant research gap. This underrepresentation may reflect deeper barriers linked to the stigmatization of abortion research and the predominant focus on crisis or humanitarian settings in studies on refugee and migrant health, where reproductive rights are often deprioritized in favor of immediate health concerns like infectious diseases and trauma. Addressing these gaps is crucial, as the barriers identified are likely just the surface of broader issues that remain unexplored. As Strong et al. have highlighted in their work on abortion stigma, addressing this issue will require confronting stigma within the academic community itself, where it manifests in the form of funding challenges or as pressure to focus on ‘less controversial’ topics [64].

## Conclusion

This review shows that refugees and migrants seeking abortion services in Europe face multiple, migration-specific access barriers across all stages of care, from limited outreach and information provision to racist discrimination during clinical encounters. Gaps in coverage for migrants/refugees without a residence permit constitute a violation of universal human rights. Improving access requires structural reforms in how abortion care is delivered. Information on availabile services must be accessible and available in relevant languages, with reliable and confidential translation services extending beyond physician appointments, and sufficient time allocated for interpreter-mediated consultations. Financial accessability will require implementing an UHC framework as well as covering indirect costs, e.g., for transportation, in advance. Safeguarding quality and safety of care further demands confronting gendered racism within health systems and centering the perspectives of refugees and migrants both in clinical practice and research. Sustainable progress, however, depends on the full de-criminalization of abortion, its recognition as a central element of reproductive health, and its integration in every national public health system. The European Commission could play a pivotal role by supporting organizations advocating for abortion rights and by promoting their inclusion in the EU Charter of Fundamental Rights.

## Supplementary Information

Supplementary Material 1.

## Data Availability

The datasets used and/or analysed during the current study are available from the corresponding author on reasonable request.

## Abbreviations

EFTA: European Free Trade Association
EP: European Parliament
EPF: European Parliamentary Forum for Sexual and Reproductive Rights
EU: European Union
GA: Gestational Age
GP: General Practitioner
IOM: International Organization for Migration
NGO: Non-governmental Organization
PCC: Population Concept Context
PRISMA: Preferred Reporting Items for Systematic reviews and Meta-Analyses
SRH: Sexual and Reproductive Health
UHC: Universal Health Coverage
UK: United Kingdom
UN: United Nations
UNHCR: United Nations High Commissioner for Refugees
WHO: World Health Organization
WoW: Women on Web
