# Supplementary material for: Understanding barriers for refugees and migrants when accessing abortion care in Europe: a scoping review
Source: BMC Public Health. 2026 Mar 21;26:1166. doi: 10.1186/s12889-026-27031-x (PMC13063723; doi:10.1186/s12889-026-27031-x)
Supplement: Supplementary file 1 — Supplementary Material 1. [file 12889_2026_27031_MOESM1_ESM.pdf]

Search strategy in PubMed

Research Question: What are barriers to **migrants' and refugees'** access to safe **abortion care** in **Europe**?

Concept 1: Migrants and refugees

Keywords
refugee* [tw] OR asylum-seeker* [tw] OR migrant* [tw] OR "undocumented person*" [tw] OR "people on the move" [tw] OR "unauthorized person*" [tw]

Mesh
"Transients and Migrants"[Mesh]
"Refugees"[Mesh]
"Refugee Camps"[Mesh]
"Abortion Applicants"[Mesh]
"Emigrants and Immigrants"[Mesh]
"Undocumented Immigrants"[Mesh]
"Medically Uninsured"[Mesh]
"Pregnant People"[Mesh]

Search block 1
"Transients and Migrants"[Mesh] OR "Refugees"[Mesh] OR "Refugee Camps"[Mesh] OR "Abortion Applicants"[Mesh] OR "Emigrants and Immigrants"[Mesh] OR "Undocumented Immigrants"[Mesh] OR "Medically Uninsured"[Mesh] OR "Pregnant People"[Mesh] OR refugee* [tw] OR asylum-seeker* [tw] OR migrant* [tw] OR "undocumented person*" [tw] OR "people on the move" [tw] OR "unauthorized person*" [tw]

Concept 2: Abortion care

Keywords
"family planning" [tw] OR abortion [tw] OR "reproductive healthcare" [tw] OR "reproductive health" [tw] OR "reproductive healthcare" [tw] OR "termination of pregnancy" [tw] OR SRH [tw] OR SRHR [tw]

Mesh
"Abortion, Induced"[Mesh]
"Reproductive Health"[Mesh]
"Sex Education"[Mesh]
"Family Planning Services"[Mesh]
"Sexual Health"[Mesh]
"Prenatal Care"[Mesh]

Search block 2
"Abortion, Induced"[Mesh] OR "Reproductive Health"[Mesh] OR "Sex Education"[Mesh] OR "Family Planning Services"[Mesh] OR "Sexual Health"[Mesh] OR "Prenatal Care"[Mesh] OR "family planning" [tw] OR abortion [tw] OR "reproductive healthcare" [tw] OR "reproductive health" [tw] OR "reproductive healthcare" [tw] OR "termination of pregnancy" [tw] OR SRH [tw] OR SRHR [tw]

Concept 3: Europe (defined as EU, EEFTA and UK)

Keywords

MesH
"Europe"[Mesh]
"Austria"[Mesh]
"Balkan Peninsula"[Mesh]
"Belgium"[Mesh]
"Europe, Eastern"[Mesh]
"Baltic States"[Mesh
"Latvia"[Mesh]]
"Estonia"[Mesh]
"Lithuania"[Mesh]
"Bosnia and Herzegovina"[Mesh]
"Bulgaria"[Mesh]
"Croatia"[Mesh]
"Czech Republic"[Mesh]
"Hungary"[Mesh]
"Poland"[Mesh]
"Romania"[Mesh]
"Slovakia"[Mesh]
"Slovenia"[Mesh]
"European Alpine Region"[Mesh]
"France"[Mesh]
"Germany"[Mesh]
"Greece"[Mesh]
"Ireland"[Mesh]
"Italy"[Mesh]
"Sicily"[Mesh]
"Luxembourg"[Mesh]
"Mediterranen Region"[Mesh]
"Mediterranean Islands"[Mesh]
"Cyprus"[Mesh]
"Malta"[Mesh]
"Netherlands"[Mesh]
"Portugal"[Mesh]
"Scandinavian and Nordic Countries"[Mesh]
"Denmark"[Mesh]
"Finland"[Mesh]
"Iceland"[Mesh]
"Norway"[Mesh]
"Sweden"[Mesh]
"Spain"[Mesh]
"Switzerland"[Mesh]
"United Kingdom"[Mesh]

Search block 3
"Europe"[Mesh] OR "Austria"[Mesh] OR "Balkan Peninsula"[Mesh] OR "Belgium"[Mesh] OR "Europe, Eastern"[Mesh] OR "Baltic States"[Mesh] OR "Latvia"[Mesh] OR

"Estonia"[Mesh] OR "Lithuania"[Mesh] OR "Bosnia and Herzegovina"[Mesh] OR "Bulgaria"[Mesh] OR "Croatia"[Mesh] OR "Czech Republic"[Mesh] OR "Hungary"[Mesh] OR "Poland"[Mesh] OR "Romania"[Mesh] OR "Slovakia"[Mesh] OR "Slovenia"[Mesh] OR "European Alpine Region"[Mesh] OR "France"[Mesh] OR "Germany"[Mesh] OR "Greece"[Mesh] OR "Ireland"[Mesh] OR "Italy"[Mesh] OR "Sicily"[Mesh] OR "Luxembourg"[Mesh] OR "Mediterranean Region"[Mesh] OR "Mediterranean Islands"[Mesh] OR "Cyprus"[Mesh] OR "Malta"[Mesh] OR "Netherlands"[Mesh] OR "Portugal"[Mesh] OR "Scandinavian and Nordic Countries"[Mesh] OR "Denmark"[Mesh] OR"Finland"[Mesh] OR"Iceland"[Mesh] OR "Norway"[Mesh] OR "Sweden"[Mesh] OR "Spain"[Mesh] OR "Switzerland"[Mesh] OR "United Kingdom"[Mesh]

"Europe"[Mesh] OR Europe [tw] OR "Austria"[Mesh] OR Austria [tw] OR "Balkan Peninsula"[Mesh] OR “Balkan Peninsula” [tw] "Belgium"[Mesh] OR Belgium [tw] OR "Europe, Eastern"[Mesh] OR “Eastern Europe” [tw] OR "Baltic States"[Mesh] OR “Baltic States” [tw] OR "Latvia"[Mesh] OR Latvia [tw] OR "Estonia"[Mesh] OR Estonia [tw] OR "Lithuania"[Mesh] OR Lithuania [tw] OR "Bosnia and Herzegovina"[Mesh] OR “Bosnia and Herzegovina” [tw] OR "Bulgaria"[Mesh] OR Bulgaria [tw] OR "Croatia"[Mesh] OR Croatia [tw] OR "Czech Republic"[Mesh] OR “Czech Republic” [tw] OR "Hungary"[Mesh] OR Hungary [tw] OR "Poland"[Mesh] OR Poland [tw] OR "Romania"[Mesh] OR Romania [tw] OR "Slovakia"[Mesh] OR Slovakia [tw] OR "Slovenia"[Mesh] OR Slovenia [tw] OR "European Alpine Region"[Mesh] OR "France"[Mesh] OR France [tw] OR "Germany"[Mesh] OR Germany [tw] OR "Greece"[Mesh] OR Greece [tw] OR "Ireland"[Mesh] OR Ireland [tw] OR "Italy"[Mesh] OR Italy [tw] OR "Sicily"[Mesh] OR Sicily [tw] OR "Luxembourg"[Mesh] OR Luxembourg [tw] OR "Mediterranean Region"[Mesh] OR "Mediterranean Islands"[Mesh] OR "Cyprus"[Mesh] OR Cyprus [tw] OR "Malta"[Mesh] OR Malta [tw] OR "Netherlands"[Mesh] OR Netherlands [tw] OR "Portugal"[Mesh] OR Portugal [tw] OR "Scandinavian and Nordic Countries"[Mesh] OR "Denmark"[Mesh] OR Denmark [tw] OR "Finland"[Mesh] OR Finland [tw] OR "Iceland"[Mesh] OR Iceland [tw] OR "Norway"[Mesh] OR Norway [tw] OR "Sweden"[Mesh] OR Sweden [tw] OR "Spain"[Mesh] OR Spain [tw] OR "Switzerland"[Mesh] OR Switzerland [tw] OR "United Kingdom"[Mesh] OR “United Kingdom” [tw]

Search strategy in PubMed

**#1**	"Transients and Migrants"[Mesh] OR "Refugees"[Mesh] OR "Refugee Camps"[Mesh] OR "Abortion Applicants"[Mesh] OR "Emigrants and Immigrants"[Mesh] OR "Undocumented Immigrants"[Mesh] OR "Medically Uninsured"[Mesh] OR "Pregnant People"[Mesh] OR refugee* [tw] OR asylum-seeker* [tw] OR migrant* [tw] OR “undocumented person*” [tw] OR “people on the move” [tw] OR “unauthorized person*” [tw]
**#2**	"Abortion, Induced"[Mesh] OR "Reproductive Health"[Mesh] OR "Sex Education"[Mesh] OR "Family Planning Services"[Mesh] OR "Sexual Health"[Mesh] OR "Prenatal Care"[Mesh] OR “family planning” [tw] OR abortion [tw] OR “reproductive healthcare” [tw] OR “reproductive health” [tw] OR “reproductive healthcare” [tw] OR “termination of pregnancy” [tw] OR SRH [tw] OR SRHR [tw]
**#3**	"Europe"[Mesh] OR Europe [tw] OR "Austria"[Mesh] OR Austria [tw] OR "Balkan Peninsula"[Mesh] OR “Balkan Peninsula” [tw] "Belgium"[Mesh] OR Belgium [tw] OR "Europe, Eastern"[Mesh] OR “Eastern Europe” [tw] OR

	"Baltic States"[Mesh] OR "Baltic States" [tw] OR "Latvia"[Mesh] OR Latvia [tw] OR "Estonia"[Mesh] OR Estonia [tw] OR "Lithuania"[Mesh] OR Lithuania [tw] OR "Bosnia and Herzegovina"[Mesh] OR "Bosnia and Herzegovina" [tw] OR "Bulgaria"[Mesh] OR Bulgaria [tw] OR "Croatia"[Mesh] OR Croatia [tw] OR "Czech Republic"[Mesh] OR "Czech Republic" [tw] OR "Hungary"[Mesh] OR Hungary [tw] OR "Poland"[Mesh] OR Poland [tw] OR "Romania"[Mesh] OR Romania [tw] OR "Slovakia"[Mesh] OR Slovakia [tw] OR "Slovenia"[Mesh] OR Slovenia [tw] OR "European Alpine Region"[Mesh] OR "France"[Mesh] OR France [tw] OR "Germany"[Mesh] OR Germany [tw] OR "Greece"[Mesh] OR Greece [tw] OR "Ireland"[Mesh] OR Ireland [tw] OR "Italy"[Mesh] OR Italy [tw] OR "Sicily"[Mesh] OR Sicily [tw] OR "Luxembourg"[Mesh] OR Luxembourg [tw] OR "Mediterranean Region"[Mesh] OR "Mediterranean Islands"[Mesh] OR "Cyprus"[Mesh] OR Cyprus [tw] OR "Malta"[Mesh] OR Malta [tw] OR "Netherlands"[Mesh] OR Netherlands [tw] OR "Portugal"[Mesh] OR Portugal [tw] OR "Scandinavian and Nordic Countries"[Mesh] OR "Denmark"[Mesh] OR Denmark [tw] OR "Finland"[Mesh] OR Finland [tw] OR "Iceland"[Mesh] OR Iceland [tw] OR "Norway"[Mesh] OR Norway [tw] OR "Sweden"[Mesh] OR Sweden [tw] OR "Spain"[Mesh] OR Spain [tw] OR "Switzerland"[Mesh] OR Switzerland [tw] OR "United Kingdom"[Mesh] OR "United Kingdom" [tw]
**#4**	#1 AND #2 AND #3

Combine building blocks in query box: #1 AND #2 AND #3

Since 2014
